# Hydrodynamic Evaluation of a Filtering Hydrocyclone for Solid Particle/Water Separation

**DOI:** 10.3390/membranes14080171

**Published:** 2024-08-06

**Authors:** Daniel C. M. Cavalcante, Hortência L. F. Magalhães, Severino R. Farias Neto, Ricardo S. Gomez, João M. P. Q. Delgado, Antonio G. B. Lima, Danielle B. T. Vasconcelos, Márcio J. V. Silva, Daniel O. Farias, Suelyn F. A. M. Queiroz, Antonio C. Q. Santos, Thâmmara L. H. Tito, Emmanuel F. M. Silva

**Affiliations:** 1 Federal Institute of Education, Science and Technology of the Sertão Pernambuco, Serra Talhada 56915-899, Pernambuco, Brazil; daniel.cesar@ifsertao-pe.edu.br; 2 Science and Technology Institute, Federal University of the Vales do Jequitinhonha and Mucuri, Diamantina 39100-000, Minas Gerais, Brazil; hortencia.luma@ufvjm.edu.br; 3 Department of Chemical Engineering, Federal University of Campina Grande, Campina Grande 58429-900, Paraíba, Brazil; severino.rodrigues@ufcg.edu.br; 4 Department of Mechanical Engineering, Federal University of Campina Grande, Campina Grande 58429-900, Paraíba, Brazil; ricardo.soares@professor.ufcg.edu.br (R.S.G.); antonio.gilson@ufcg.edu.br (A.G.B.L.); 5 CONSTRUCT-LFC, Civil Engineering Department, Faculty of Engineering, University of Porto, 4200-465 Porto, Portugal; 6 Federal Institute of Education, Science and Technology of Alagoas, Piranhas 57460-000, Alagoas, Brazil; danielle.silva@ifal.edu.br; 7 Federal Institute of Education, Science and Technology of Pernambuco, Vitória de Santo Antão 56600-000, Pernambuco, Brazil; marcio.vasconcelos@vitoria.ifpe.edu.br; 8 Department of Production Engineering, Federal University of Campina Grande, Sumé 58540-000, Paraiba, Brazil; daniel.oliveira@professor.ufcg.edu.br; 9 CERTBIO, Department of Materials Engineering, Federal University of Campina Grande, Campina Grande 58429-900, Paraíba, Brazil; morais.suelyn@gmail.com (S.F.A.M.Q.); antoniocarlos_queiroz@hotmail.com (A.C.Q.S.); thammaratito@gmail.com (T.L.H.T.); 10 Department of Agricultural Engineering, Federal University of Campina Grande, Campina Grande 58429-900, Paraíba, Brazil; emmanuel.atercg@gmail.com

**Keywords:** hydrocyclones, fluid dynamics, porous membrane, Ansys CFX

## Abstract

A conventional hydrocyclones is a versatile equipment with a high processing capacity and low maintenance cost. Currently, several studies aim to alter the typical structure of the conventional hydrocyclone in order to modify its performance and purpose. For this, filtering hydrocyclones have emerged, where a porous membrane replaces the conic or cylindrical wall. During the operation of this equipment, in addition to the traditionally observed streams (feed, underflow, and overflow), there is a liquid stream resulting from the filtration process, commonly referred to as filtrate. This work proposes to numerically investigate the solid particle/liquid water separation process in a filtering hydrocyclone using the commercial software Ansys CFX^®^ 15.0. The proposed mathematical model for the study considers three-dimensional, steady state and turbulent flow, using the Eulerian–Eulerian approach and the Shear Stress Transport (SST) turbulence model. This study presents and analyzes the volume fraction, velocity, and pressure fields, along with flowlines and velocity profiles. The results indicate that the proposed model effectively captures the fluid dynamic behavior within the filtering hydrocyclone, highlighting higher pressures near the porous membrane and a higher concentration of solid particles in the conical region, with water being more concentrated in the cylindrical part of the hydrocyclone. Additionally, the findings show that the volumetric flow rate of the filtrate significantly influences the internal flow dynamics, with conventional hydrocyclones demonstrating higher pressure gradients compared to the proposed filtering hydrocyclone.

## 1. Introduction

One of the major challenges in the petroleum industry is the treatment of its effluents before disposal into the environment. A significant portion of these waste materials consist of solid particles and/or liquids that generally need to be treated for proper disposal or even for reuse. Oil pollution is a highly sensitive issue; its presence in water bodies negatively impacts aeration and light penetration in water courses, creating an insoluble film on the water surface, which produces harmful effects on fauna and flora [1,2].

Oily waters refer to a water–oil mixture originating from the petroleum industry, which can be observed at different stages of production, extraction, transportation, and refining. The shearing caused by pumps, valves, accessories (curves, bifurcations, etc.), and other equipment leads to a mixing of phases (water and oil) and often results in emulsions. In order to minimize these effects, many studies have been conducted on water/oil or water/oil/solid particle separation processes, employing, for example, processes that use circular or swirling movements induced by one or more tangential inlets, such as the hydrocyclone [3,4,5,6,7,8,9,10,11,12,13,14,15].

Hydrocyclones are devices generally equipped with one or more tangential inlets perpendicular to a cylindrical section connected to a conical section. Within the cylindrical section, there is also a central tube. Hydrocyclones are categorized into families based on their shape and geometric dimensions, which play a crucial role in the separation process, as these factors are directly related to processing capacity and separation efficiency.

Hydrocyclone families with a relatively large cylindrical region exhibit higher processing capacity, while those with a larger dimension in the conical part induce greater collection efficiency. Generally, hydrocyclones have a conical section larger than the cylindrical one [3,4,14,16,17,18].

Due to the good technical and economic characteristics of hydrocyclones, their application in various physical problems in industrial and academic areas has gained significant attention [19,20,21,22,23,24,25,26,27].

The evolving landscape of hydrocyclone technology has seen significant advancements with the advent of numerical methods that aid in the design, operation, and optimization of these devices. Traditionally, hydrocyclone performance has been analyzed through empirical and mechanistic models, each offering distinct advantages [19,20]. Empirical models, derived from extensive regression analysis, provide quick and reliable predictions under specific conditions but often fail to capture the complexities of hydrocyclone operations outside those conditions. Mechanistic models, on the other hand, leverage fundamental physical principles to simulate detailed flow fields and separation processes, although their computational intensity makes them less practical for real-time industrial applications.

Numerical methods, including computational fluid dynamics (CFDs), have become indispensable tools in addressing these challenges. CFDs, in particular, allow for the detailed simulation of multiphase flow dynamics within hydrocyclones, providing insights into the interactions between solid particles and fluid phases. This method enables the analysis of complex phenomena such as turbulence, particle tracking, and phase separation, which are critical for optimizing hydrocyclone performance [21].

Regarding the hydrocyclone, the selection of a hydrocyclone family is typically based on the equipment’s requirement for high classification power or high concentrating power. Additionally, hydrocyclones can effectively separate particles ranging from 5 to 400 μm, highlighting their versatility in applications such as selective classification, thickening, fractionation, liquid recovery, and pre-concentration, among others [22].

Vieira [22] conducted an investigation and optimization of filtering hydrocyclones performance using geometric relationships derived from experimental design techniques. The purpose was to maximize collection efficiencies while minimizing energy costs. The study concluded that filtration significantly influences the hydrocyclone’s performance. In comparison to conventional hydrocyclones operating at the same volumetric feed flow rate, filtering hydrocyclones were capable of providing a lower pressure drop during solid–liquid separation. Furthermore, filtration reduced and dampened the spiral flow movement inside the filtering hydrocyclone.

Henrique et al. [23] proposed modifying a conventional hydrocyclone by replacing the non-porous conical section with a porous and permeable conical section, naming it a filtering hydrocyclone. Subsequently, Barrozo et al. [24] presented initial findings using a filtering hydrocyclone designed based on Bradley’s geometry with a nylon porous medium. The authors observed substantial changes in the main operational parameters, leading primarily to enhanced processing capacity and a decrease in the liquid ratio and collection efficiency.

Vieira [25] reported research on a filtering hydrocyclone based on Bradley’s geometry using polypropylene as the filtering medium. The author observed that operational variables still exhibited trends similar to those observed by Barrozo et al. [24], differing only in magnitude. In another study, Vieira [26] investigated a filtering hydrocyclone based on Reitema’s geometry, concluding that both filtration and the geometry of the conical trunk significantly influenced the fluid dynamics of the system. Additionally, it was observed that the influence of operational variables for Reitema’s filtering hydrocyclones moved in the opposite direction compared to Bradley’s filtering hydrocyclones.

Façanha [28], studying a filtering hydrocyclone with a cylindrical wall, analyzed the effect of the permeability of the porous medium. The author concluded that the presence of the filtrate flow related to the cylindrical membrane influenced the flow hydrodynamics inside the hydrocyclone, resulting in a lower Euler number and liquid ratio compared to the conventional cylindrical hydrocyclone. Additionally, the total separation efficiency of the filtering hydrocyclone was approximately 11% lower than that achieved for a conventional cylindrical hydrocyclone.

In addition to the previously mentioned works, more experimental and numerical studies on filtering hydrocyclones can be found in the literature [29,30,31,32,33,34,35,36,37,38,39,40,41,42,43,44]. However, as this is a relatively new technology, studies in this field are still limited. A better understanding of the transport phenomena associated with this type of separation equipment becomes crucial, especially for achieving higher separation efficiency. In this context, the present work aims to investigate the fluid dynamics inside a filtering hydrocyclone for solid particle/water separation. The purpose is to assist researchers, process engineers, and industrial professionals in decision-making regarding the choice and selection of these equipment for specific applications.

## 2. Methodology

### 2.1. Problem Description

**Figure 1:**
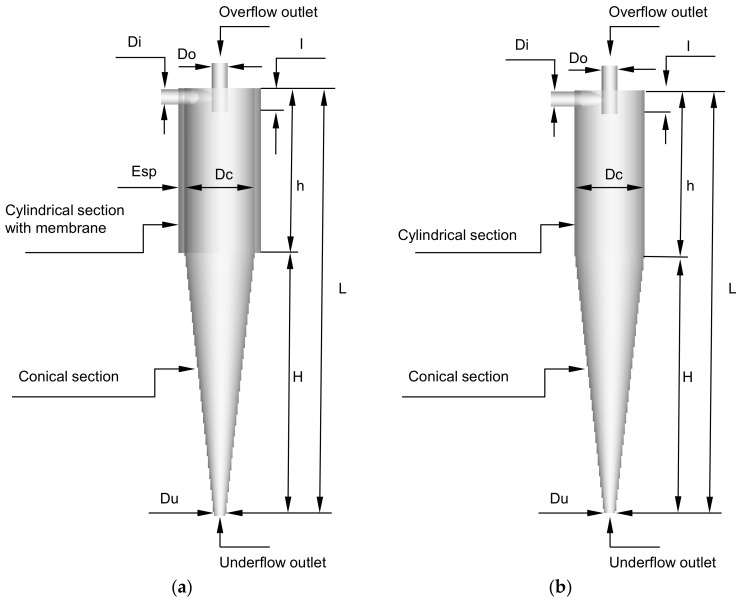
Hydrocyclones evaluated in this study: (**a**) filtering cylindrical hydrocyclone (HciF) and (**b**) conventional hydrocyclone (Hcon).

The studied hydrocyclone is based on the work of Façanha [28], featuring a porous matrix in the cylindrical part and an impermeable conical section, referred to as a filtering cylindrical hydrocyclone. Figure 1 illustrates the geometries of both the filtering and conventional hydrocyclones used in this research. Table 1 provides the dimensions of the filtering equipment, which has the same dimensions as the conventional hydrocyclone.

### 2.2. Computational Domain

The entire study was conducted using Ansys CFX 15.0 software (Canonsburg, PA, USA). The mesh generation for the hydrocyclone was carried out following the steps below:

(a) Definition of a set of points and curves to represent the geometry;
(b) Selection of a set of blocks through division, merging, boundary definitions, face modifications, and vertex movements;
(c) Verification of block quality to ensure that the blocking generated a high-quality mesh;
(d) Assessment of mesh quality.

**Figure 2:**
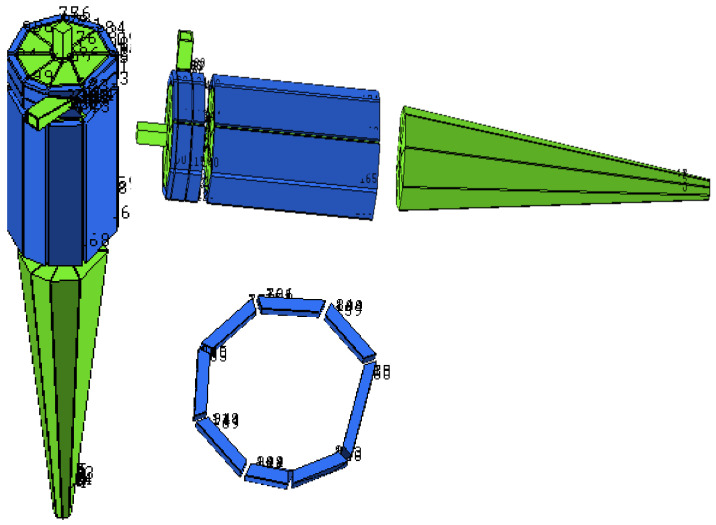
Blocking representation: filtering cylindrical hydrocyclone.

**Figure 3:**
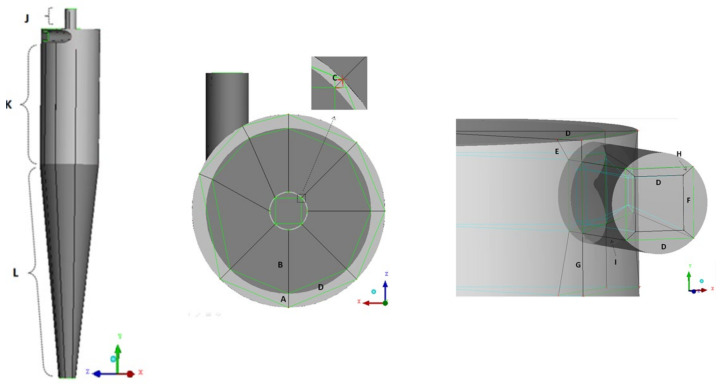
Lines (edges) used for mesh construction as per Table 3. Letters indicate regions in analysis.

Figure 2 illustrates the blocking strategy employed to generate the meshes used in the simulations of different studied cases. Figure 3 shows the edges of the blocks responsible for mesh refinement. The values of the number of divisions for each edge are listed in Table 2.

Three meshes with different control volume (element) densities were generated to conduct the mesh dependency study. The number of elements and nodes for each mesh is provided in Table 3.

**Figure 4:**
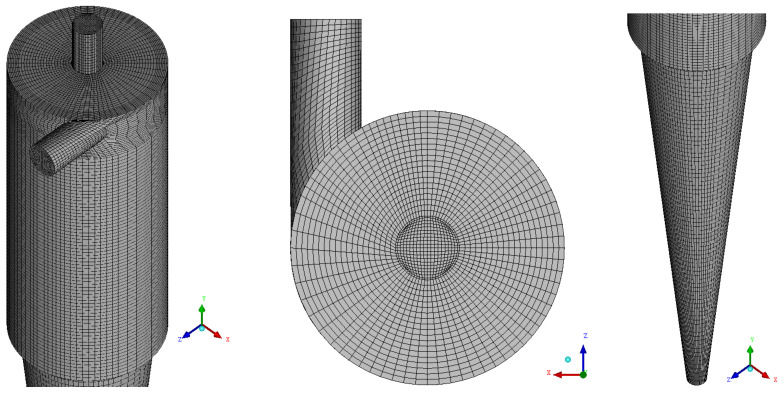
Representation of the mesh of the filtering cylindrical hydrocyclone.

Figure 4 shows mesh 03 of the filtering cylindrical hydrocyclone, with detail the refinement performed.

### 2.3. Mathematical Modeling

#### 2.3.1. The Model

The mathematical model used to describe the separation process in the filtering cylindrical hydrocyclone (HciF) is based on the Eulerian–Eulerian approach. The following simplifications were adopted:

(a) Incompressible, steady, and isothermal flow.
(b) Water as the continuous phase, and solid particles as the dispersed phase.
(c) Particles were assumed to be spherical with a diameter of 9.34 μm [28].
(d) Flow with constant physical and chemical properties.
(e) There is no mass source.
(f) Porosity and permeability were assumed to be uniformly distributed in the porous medium.

With these considerations, the conservation equations for mass and linear momentum for the two-phase flow reduce to the following:

(a) *Mass Conservation*

(1) $$\nabla .\left(f_{\alpha }\rho_{\alpha }{\vec{U}}_{\alpha }\right)=0$$

where f is the volume fraction, $\vec{U}$ is the velocity vector, and ρ is the density.

(b) *Linear Momentum Conservation*

(2) $$\nabla \cdot \left[f_{\alpha }\left(\rho_{\alpha }{\vec{U}}_{\alpha }⊗{\vec{U}}_{\alpha }\right)\right]=-f_{\alpha }\nabla p_{\alpha }+\left\{f_{\alpha }\mu_{\alpha }\left[\nabla {\vec{U}}_{\alpha }+{\left(\nabla {\vec{U}}_{\alpha }\right)}^{T}\right]\right\}+{\vec{M}}_{\alpha }+\rho \vec{g}$$

with ${\vec{M}}_{\alpha }$ (drag force) defined by the following:

(3) $${\vec{M}}_{\alpha }=\frac{3C_{d}}{4d_{p}}f_{\beta }\rho_{\alpha }\left|{\vec{U}}_{\beta }-{\vec{U}}_{\alpha }\right|\left({\vec{U}}_{\beta }-{\vec{U}}_{\alpha }\right)$$

and $C_{d}$ is determined using the Schiller–Naumann model modified as follows:

(4) $$C_{d}=max[\frac{24}{Re}\left(1+0.15Re^{0.687}\right),0.44]$$

(c) *Turbulence Model*

In this research, the SST (Shear Stress Transport) turbulence model was used for the continuous phase (water). Applying the adopted considerations, the equations of this model are reduced to the following:

(5) $$\nabla \cdot \left(\rho \vec{U}k\right)=\nabla \cdot \left[\left(\mu +\frac{\mu_{t}}{\sigma_{k2}}\right)\nabla k\right]+P_{k}-\beta ’\rho k\omega$$

(6) $$\nabla \cdot \left(\rho \vec{U}\omega \right)=\nabla \cdot \left[\left(\mu +\frac{\mu_{t}}{\sigma_{\omega 2}}\right)\nabla k\right]+\left(1-F_{1}\right)2\rho \frac{1}{\sigma_{\omega 2}\omega }\nabla k\nabla \omega +\alpha_{2}\frac{\omega }{k}P_{k}-\beta_{2}\rho \omega^{2}$$

For the dispersed phase, the zero-equation dispersion model was adopted, defined by Equation (7):

(7) $$\mu_{t,d}=\left(\frac{\rho_{d}}{\rho_{c}}\right)\frac{\mu_{t,c}}{\sigma }$$

where

(8) $$F_{1}=\tan h\left({arg}_{1}\right)$$

(9) $$\arg_{1}=\min\left[\max\left(\frac{\sqrt{k}}{\beta^{’}\varpi y^{’}},\frac{500v}{y^{2}\varpi }\right),\frac{4\rho k}{{CD}_{k\varpi }\sigma_{\varpi 2}y^{2}}\right]$$

(10) $$CD_{k\varpi }=\max\left(2\rho \frac{1}{\sigma_{w2}\varpi }\nabla k\nabla \varpi ,\;1.0\times 10^{-10}\right)$$

where y is the distance near the wall, and ν is the kinematic viscosity. The constants used in this model are given by α2=0.44;  β2=0.0828;  σϖ2=10.856 and $\;\sigma_{k2}=1$. 

The transport behavior can be obtained by a limiter to the turbulent viscosity formulation:

(11) $$\nu_{t}=\frac{\alpha_{1}k}{\max\left(\alpha_{1}\varpi ,SF_{2}\right)}$$

(12) $$\nu_{t}=\frac{\mu_{t}}{\rho }$$

where *F_2_* is a blending function, similar to *F_1_*, which determines the wall limit to the boundary layer, and *S* is an invariant measure of the rate of strain tensor.

The blending functions are critical for the success of the method. Their formulation are based on the distance near the surface and the flow variables, as follows:

(13) $$F_{2}=\tan h(\arg_{2}^{2})$$

(14) $$\arg_{2}=\max\left(\frac{2\sqrt{k}}{\beta ’\varpi y},\frac{500v}{y^{2}\varpi }\right)$$

For a better understanding, the blending functions have the characteristic of delineating the zones where each model will operate. Based on the values found for these functions, there are changes in the model equations, where blending function *F_1_* is responsible for switching models in the transport equations for ω and k, determining the model constants. Meanwhile, blending function *F_2_* is responsible for switching models in the turbulent viscosity formulation.

(d) *Porous Medium Model*

The conservation equations for multiphase flow for the cylindrical membrane:

(15) $$\nabla \cdot \left(f_{\alpha }\rho_{\alpha }K{\vec{U}}_{\alpha }\right)=0$$

where $K=(K^{ij})$ is a second-order symmetric tensor, called the permeability tensor.

(16) $$\nabla \cdot \left[f_{\alpha }\rho_{\alpha }\;\left(K\cdot \vec{U}\right)⊗\vec{U}\right]=-\nabla p+\nabla \cdot \left\{f_{\alpha }\mu_{e}K\cdot \left[\nabla \vec{U}+{\left(\nabla {\vec{U}}_{i}\right)}^{T}\right]\right\}+S_{i}^{M}$$

where $\mu_{e}$ is the effective viscosity defined by the following equation:

(17) $$\mu_{e}=\mu_{\alpha }+\mu_{t}$$

where $\mu_{\alpha }$ is the dynamic viscosity, and $\mu_{t}$ is the turbulent viscosity.

#### 2.3.2. Boundary Conditions Used in Simulations

(a) *Inlet*

A prescribed volumetric flow rate condition was applied at the inlet region.

(b) *Walls*

For the fluid phases, no-slip wall conditions were used, meaning that the fluid near the wall has a velocity of zero. Thus, by definition:

(18) $${\vec{U}}_{\alpha }=0$$

where ${\vec{U}}_{\alpha }$ is the velocity vector of the water phase.

For the particulate phase, a free-slip condition was applied, which is used when the shear stress at the wall is zero, and the fluid velocity near the wall is not reduced by the effect of friction. It is given by the following equation:

(19) $$\frac{\partial u_{s}}{\partial r}=\frac{\partial v_{s}}{\partial r}=\frac{\partial w_{s}}{\partial r}=0$$

where $u_{s}$, $v_{s},$ and $w_{s}$ are the components of the solid phase velocity vector.

(c) *Outlets*

At the upper and lower outlet sections of the hydrocyclone, a prescribed average static pressure equal to atmospheric pressure was used.

#### 2.3.3. Thermophysical and Chemical Parameters of Materials Used in Simulations

The physicochemical properties for the liquid and solid phases at room temperature of 293.15 K used in this work are presented in Table 4 and Table 5.

#### 2.3.4. Auxiliary Equations

(a) *Pressure Drop*

The pressure drop ∆P is calculated considering the pressure at the inlet and at the upper outlet or “overflow”.

(20) $$∆P=P_{inlet}-P_{overflow}$$

(b) *Filtrate Flow*

The filtrate flow is calculated by the ratio of the mass flow fraction of the fluid to the water density, given by the following equation:

(21) $$Q_{F}=\frac{\dot{m}}{\rho_{water}}$$

(c) *Euler Number*

**Figure 5:**
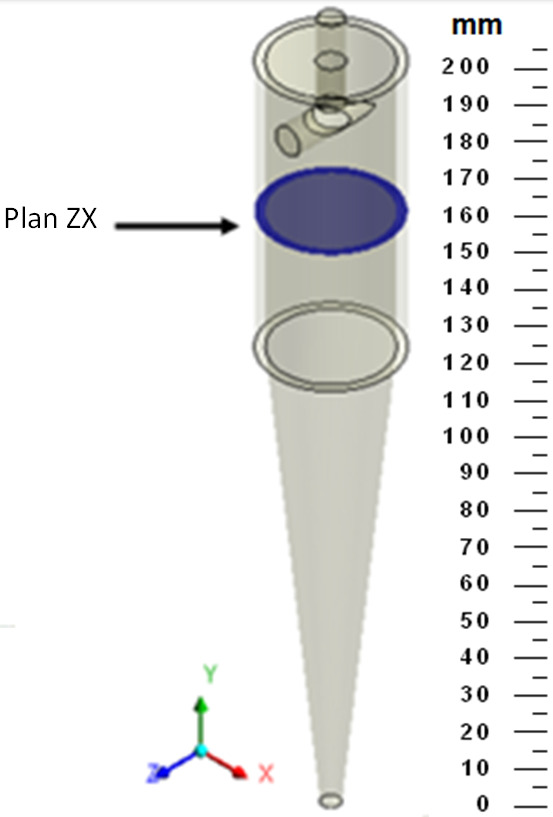
Representation of the ZX plane (Y=0.165 mm) inside the hydrocyclone.

The Euler number was calculated with the aid of the ZX cross-sectional plane at a position y=165 mm inside the hydrocyclone (Figure 5). The mass flow rate was determined on this plane.

From the mass flow rate value on the plane, the Euler number was then determined using the following equation:

(22) $$Eu=-\frac{∆P}{\rho u_{c}^{2}/2}$$

where

(23) $$u_{c}=4\frac{\dot{m}}{\pi \rho_{water}D_{C}^{2}}$$

(d) *Liquid Ratio*

The liquid ratio was calculated from the volumetric inflow rates $Q_{a}$ and the lower outlet (“underflow”) $Q_{u}$, defined by the following equation:

(24) $$R_{L}=1-\frac{Q_{u}}{Q_{a}}$$

(e) *Total Efficiency*

The total efficiency was calculated from the inlet mass flow rate $W_{a}$ and the lower outlet (underflow) $W_{u}$, defined by the following equation:

(25) $$\eta =\frac{W_{u}}{W_{a}}$$

### 2.4. Studied Case

For the analysis of the flow dynamics in the hydrocyclone and the mesh study, the conditions presented in Table 6 were used.

## 3. Results and Discussion

### 3.1. Mesh Quality Assessment

The mesh created for this study is one of the factors influencing the quality of numerical results, which should be independent of the element density, the ratio between the size of each element and the reference element (expansion factor), the quantity of tetrahedral and/or hexahedral elements, mesh refinement, among others. An inadequate mesh for a study negatively impacts the solution’s accuracy, the required simulation time, and the convergence (or divergence) rate of results, among other numerical aspects.

**Figure 6:**
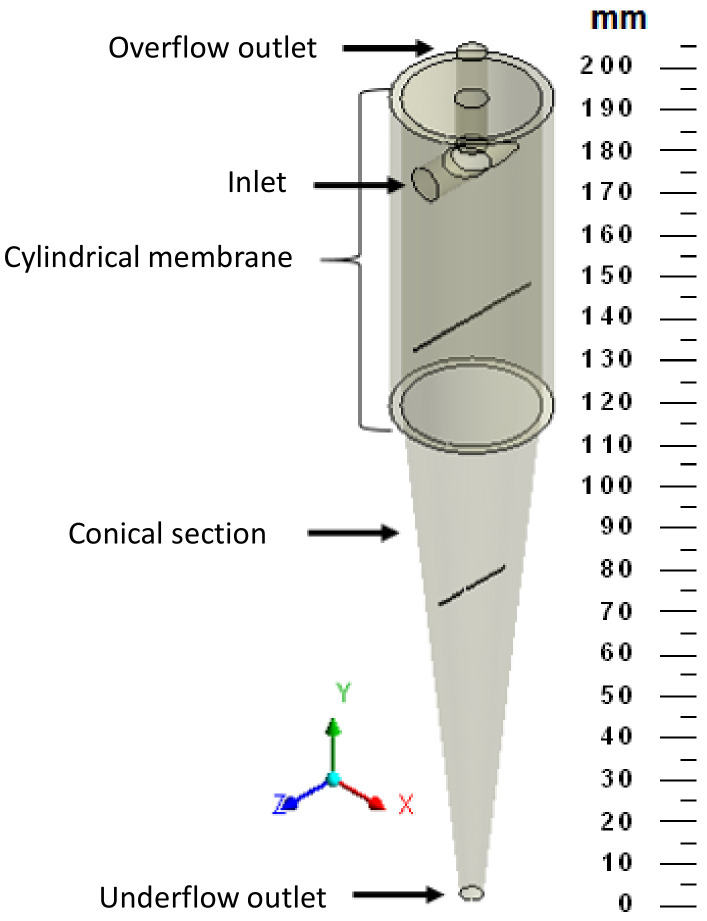
Hydrocyclone with cylindrical filtering medium.

In this research, studies were conducted with the three meshes listed in Table 3 to evaluate the influence of mesh resolution on the results of the filtering cylindrical hydrocyclone. Fluid velocity results were collected along two lines: one at y=150 mm (cylindrical part) and the other at y=80 mm (conical part), as illustrated in Figure 6. 

**Figure 7:**
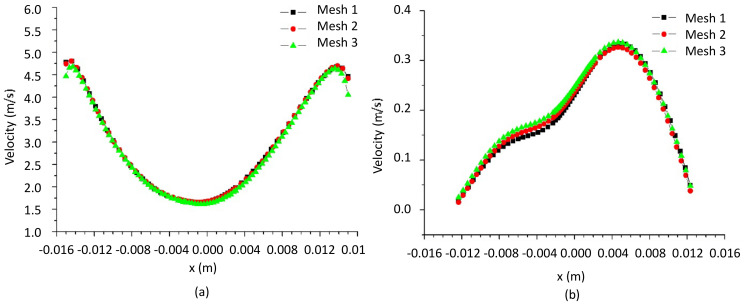
The resultant fluid velocity as a function of the z-position, at (**a**) Y=150 mm and (**b**) Y=80 mm, for the three numerical meshes.

Figure 7 illustrates the fluid velocity along the z-position at y values of (a) 150 mm and (b) 80 mm, respectively, for the three developed meshes. It can be observed that the fluid velocity exhibits the same behavior for all three meshes, indicating that the constructed meshes do not influence the results. Furthermore, the similar behavior of the velocity profiles suggests that the results are not affected by mesh refinement at the levels used in this study. Therefore, due to the independence of the results from the analyzed meshes and having an intermediate level of refinement, mesh 02 was used in the subsequent simulations.

### 3.2. Flow Dynamics in the Hydrocyclone with Cylindrical Filtering Medium

Due to the limited number of studies in the literature on CFD applied to hydrocyclones containing the cylindrical part as a filter and the high complexity of the flow inside these devices, this research aims to contribute to this area, seeking to better understand the behavior of phases during the separation process in this type of equipment.

For the validation of this research, the predicted results of the hydrodynamic parameters obtained herein were compared with the experimental results reported by Façanha [28]. From the numerical results, a pressure drops of 88 kPa was obtained in the filtering hydrocyclone, resulting in a predicted filtrate flow rate of 0.053 cm^3^/s, a Euler number of 1049, a liquid ratio of 32.4, and a separation efficiency of 73.1%. The experimental results for these same parameters reported by Façanha [28] were 88 kPa for the pressure drop, resulting in an error of 0.0%; 0.052 cm^3^/s for the filtrate flow rate, corresponding to an error of 1.9%; 1012 for the Euler number, resulting in an error of 3.6%; and 32.4 for the liquid ratio, representing an error of 0.0%, and 74.9% for the separation efficiency, resulting in an error of 2.4%, which can be considered small given the complexity of the studied phenomenon.

**Figure 8:**
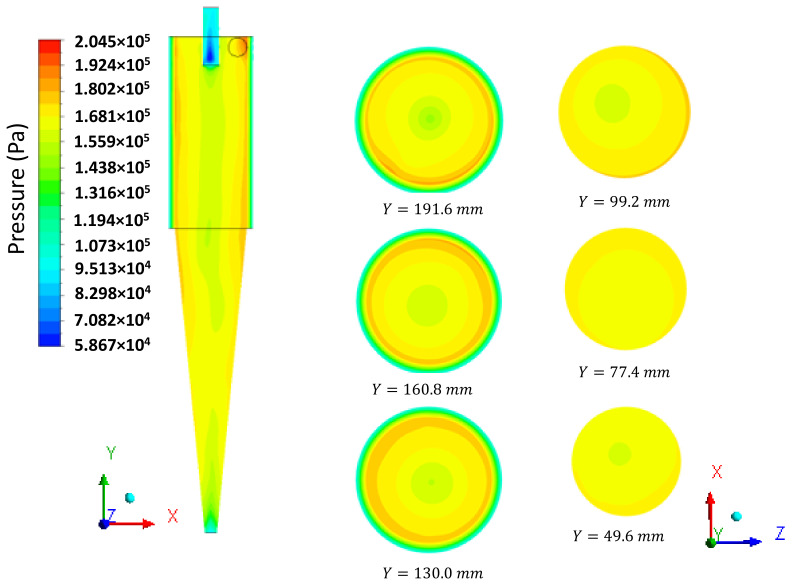
Pressure field on the XY and XZ planes inside the filtering cylindrical hydrocyclone.

**Figure 9:**
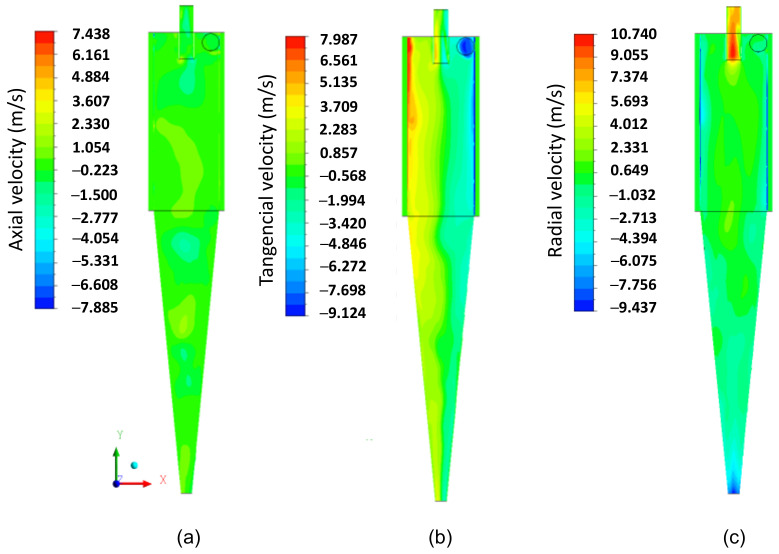
(**a**) Axial, (**b**) tangential, and (**c**) radial velocity fields on the XY plane inside the filtering cylindrical hydrocyclone.

Subsequently, we present an in-depth discussion about different hydrodynamic parameters. In Figure 8, the pressure field inside the hydrocyclone is represented in the longitudinal YZ and transversal XZ planes at different positions: Y=191.6 mm, 160.8 mm, 130mm, 99.2 mm, 77.4 mm, and 49.6 mm. It is observed that the pressure in the cylindrical part increases radially from the center to the wall, i.e., there are regions of low pressure near the central axis of the hydrocyclone and regions with higher pressures near the walls due to the centrifugal force caused by tangential velocity. This behavior is reaffirmed in Figure 9b, where the tangential velocity field inside the HciF is represented in the XY plane with a feed flow rate of 295.7 cm³/s. These results are similar to those observed in conventional and conical filtering hydrocyclones, as reported in the literature [22,35,36,37,38].

In Figure 9a–c, the axial, tangential, and radial velocity fields on the XY plane, respectively, are represented for the filtering cylindrical hydrocyclone (HciF) with volumetric feed flow 295.7 cm³/s. The combination of tangential and radial velocities generates regions of low pressure inside the hydrocyclone, reaching values close to atmospheric pressure in the central region.

A comparison between Figure 9a,c reveals that the filtration process influenced the characteristics of the axial and radial fluid velocity, confirming the behavior reported in the literature that the radial velocity of the fluid increases from the wall to the axis of the hydrocyclone [14,22,45]. These velocity components induce particles to remain in the external vortex, leading them to exit the hydrocyclone through the lower outlet or “underflow”.

**Figure 10:**
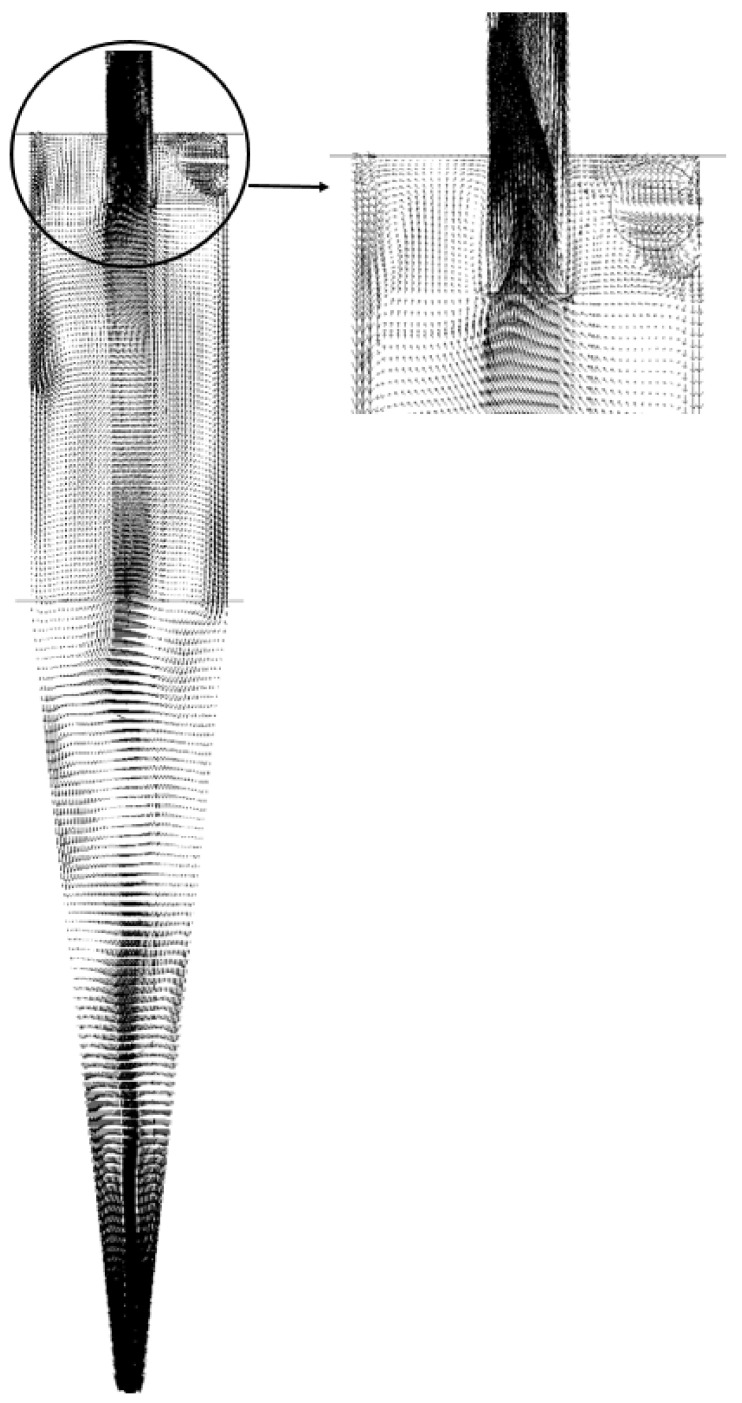
Vector field of the flow inside the filtering cylindrical hydrocyclone.

Upon analyzing Figure 10, which illustrates the distribution of axial velocity components inside the hydrocyclone, a noticeable decrease in velocity is observed from the wall towards the center of the hydrocyclone, consistent with findings reported by Oliveira et al. [38]. Furthermore, it is noted that the velocities have negative values, indicating recirculation zones. Throughout both the cylindrical and conical parts, it is observed that the recirculation region is more pronounced when there is an increase in radial velocity and a decrease in tangential velocity. This behavior is more accentuated in the conical part of the hydrocyclone, where the radial velocity is higher.

In Figure 9b, it can be observed that the tangential velocity decreases inside the cylindrical part until it reaches the transition region with the conical part, where a significant decrease in tangential velocity occurs, being overcome by radial and axial velocities upon reaching the conical part, due to the progressive decrease in radius towards the ‘underflow’ outlet. As the fluid moves away from the hydrocyclones’ inlet section, the tangential velocity decreases. This may be a possible explanation for the fluid behavior shown in Figure 9b, caused by the filtrating cylindrical wall, due to the additional outflow, i.e., the filtrate. The corresponding filtrate flow rate was 0.050 cm³/s, which is much smaller than that observed in the overflow and underflow outlets, but sufficient to make the flow inside the hydrocyclone quite complex.

In Figure 10, the velocity vector field of the fluid is reported on the longitudinal XY plane for the HciF with a volumetric feed flow rate of 295.7 cm³/s. A chaotic flow pattern is observed in the upper part of the hydrocyclone, known as short circuit or by-pass according to Silva [40], Façanha [28], and Salvador et al. [42]. This flow formation arises due to friction in the upper part of the hydrocyclone, which locally decelerates the flow. As the fluid collides with the wall, it creates a region of chaotic flow near the external wall of the vortex finder, considerably decreasing the material flow. Consequently, a portion of the fluid deviates from the preferred path and, thus, bypasses the separation process, regardless of particle size or density. This phenomenon has been widely observed in various types of hydrocyclones, as reported in the literature [22,28,35,40,42,44,46].

**Figure 11:**
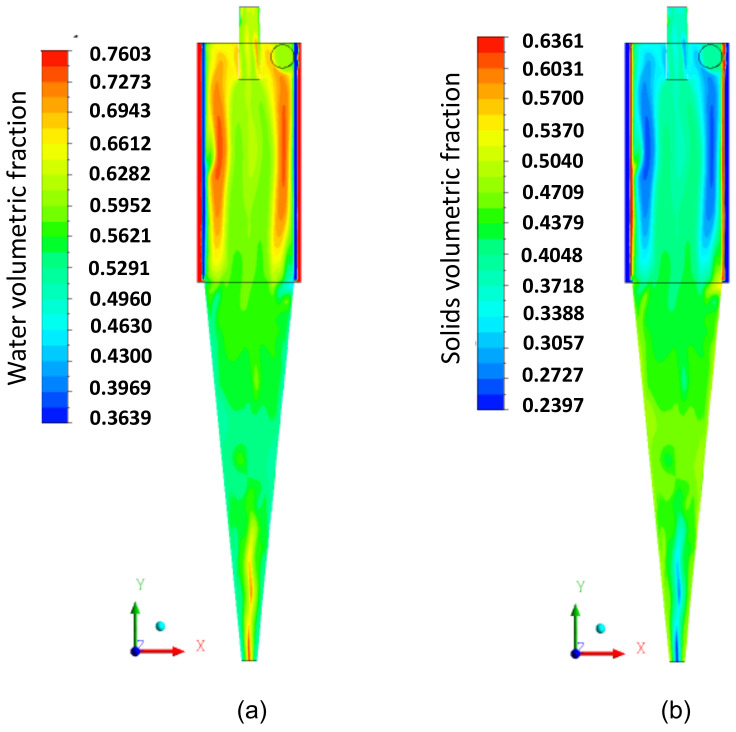
The volumetric fraction field of water (**a**) and solid particles (**b**) on the XY plane inside the filtering cylindrical hydrocyclone.

**Figure 12:**
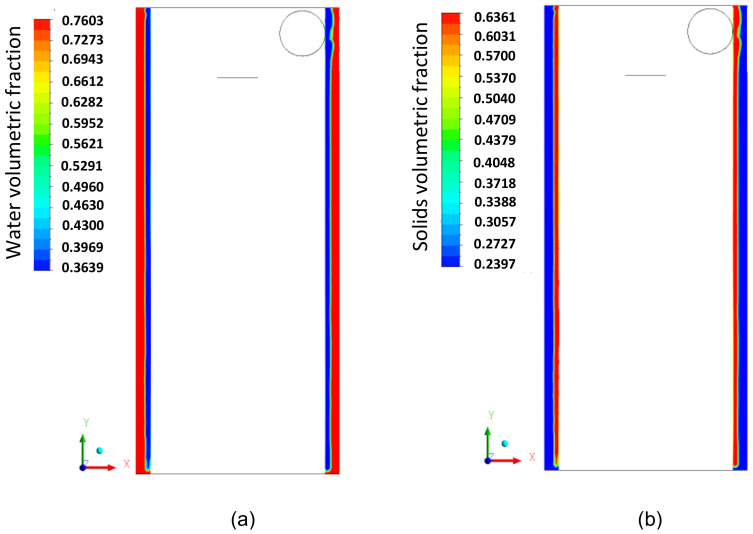
The volumetric fraction field of water (**a**) and solid particles (**b**) for the cylindrical membrane on the XY plane at the wall of the filtering cylindrical hydrocyclone.

Figure 11a,b illustrate the volume fraction field on the longitudinal XY plane for the phases (a) fluid and (b) solid particles. It is observed that there is a tendency for a higher concentration of particles in the conical part of the hydrocyclone (Figure 11b) and water in the cylindrical part (Figure 11a). This occurs due to the difference in density between the liquid and solid phases. Additionally, in Figure 11b, the penetration of solid particles into the interior of the cylindrical membrane is observed, which, depending on the intensity of this effect, can increase the flow resistance inside the membrane, leading to a possible partial clogging of the pores, due to the size of the particles being smaller than the pore size. This behavior is better observed in Figure 12, where the volume fraction fields of the fluid and particles in the filtering medium are represented.

### 3.3. A Comparison between the Conventional and Filtering Hydrocyclones

In this section, the flow behavior inside a filtering cylindrical hydrocyclone is evaluated and compared with a conventional hydrocyclone, both with the same geometry and volumetric flow rate of 295.7 cm³/s.

**Figure 13:**
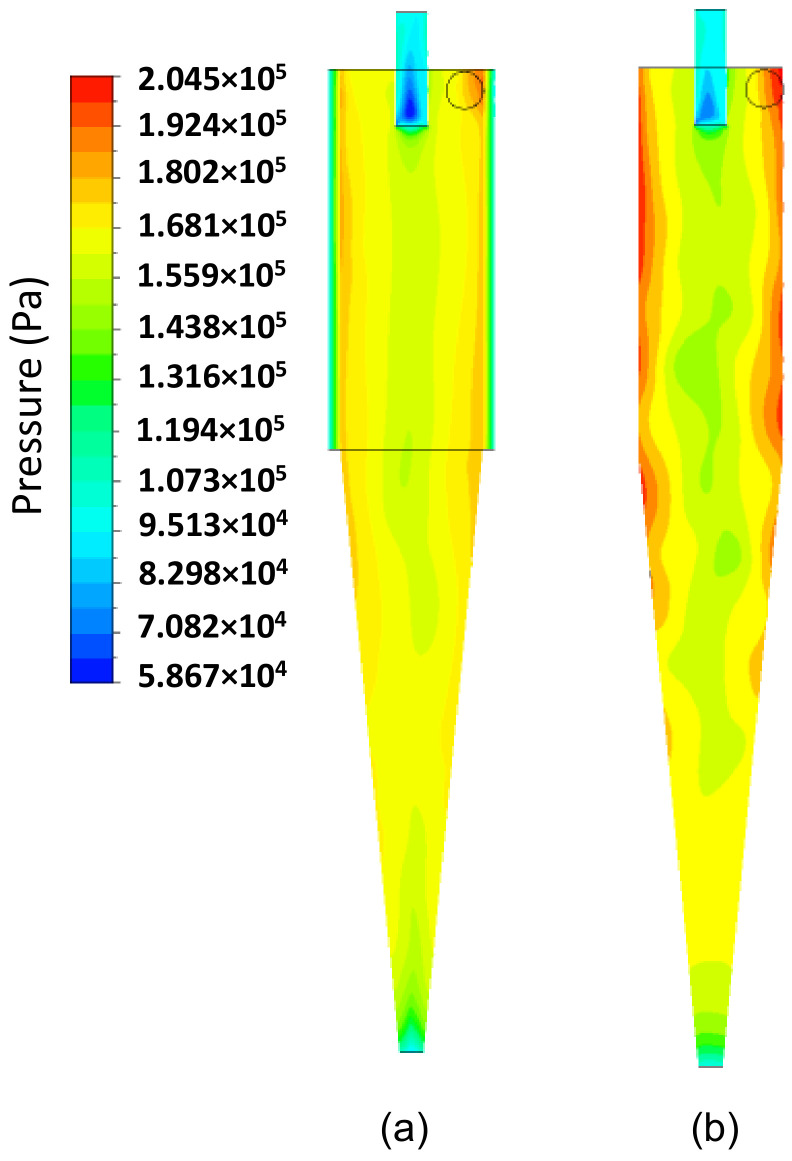
The pressure field on the XY plane for (**a**) the filtering cylindrical hydrocyclone (HciF) and (**b**) the conventional hydrocyclone (Hcon).

Figure 13 shows the pressure field in the longitudinal XY position for both the filtering hydrocyclone (HciF) and the conventional hydrocyclone (Hcon). Upon analysis, it is evident that the pressure field in the filtering hydrocyclone has a higher value near the walls, compared to the conventional hydrocyclone. This can be explained by the presence of the filtering medium, which provides a flow of filtrate and, thus, reduces pressure gradients near the cylindrical wall of the hydrocyclone. The conventional hydrocyclone exhibited a higher pressure drop than that obtained with the filtering hydrocyclone, resulting in a higher Euler number (Eu = 1092) compared to that obtained for the filtering hydrocyclone (Eu = 1012), indicating a higher energy expenditure for processing. 

**Figure 14:**
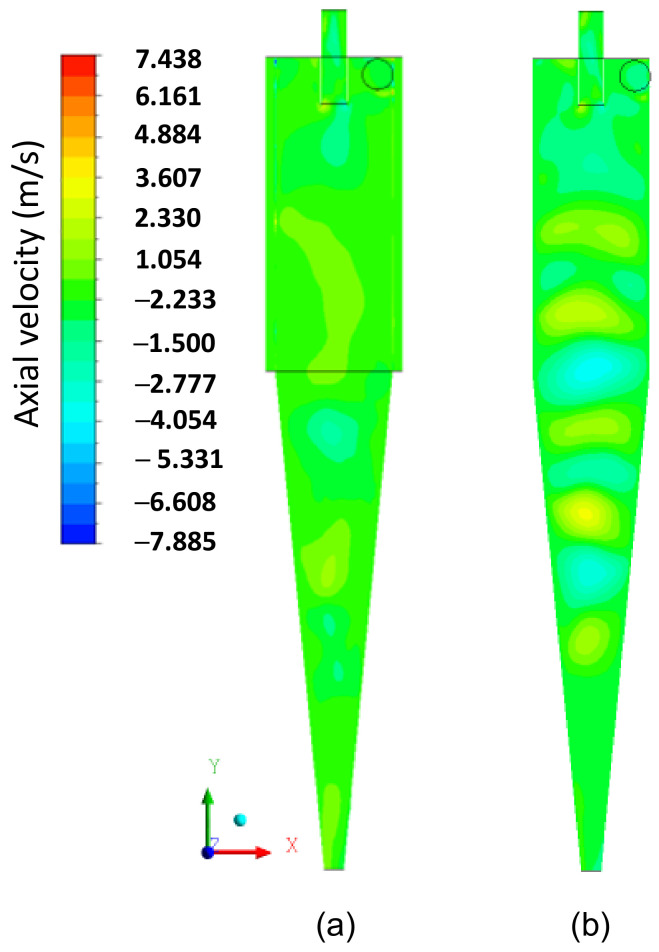
The axial velocity field on the XY plane for (**a**) the filtering cylindrical hydrocyclone (HciF) and (**b**) the conventional hydrocyclone (Hcon).

**Figure 15:**
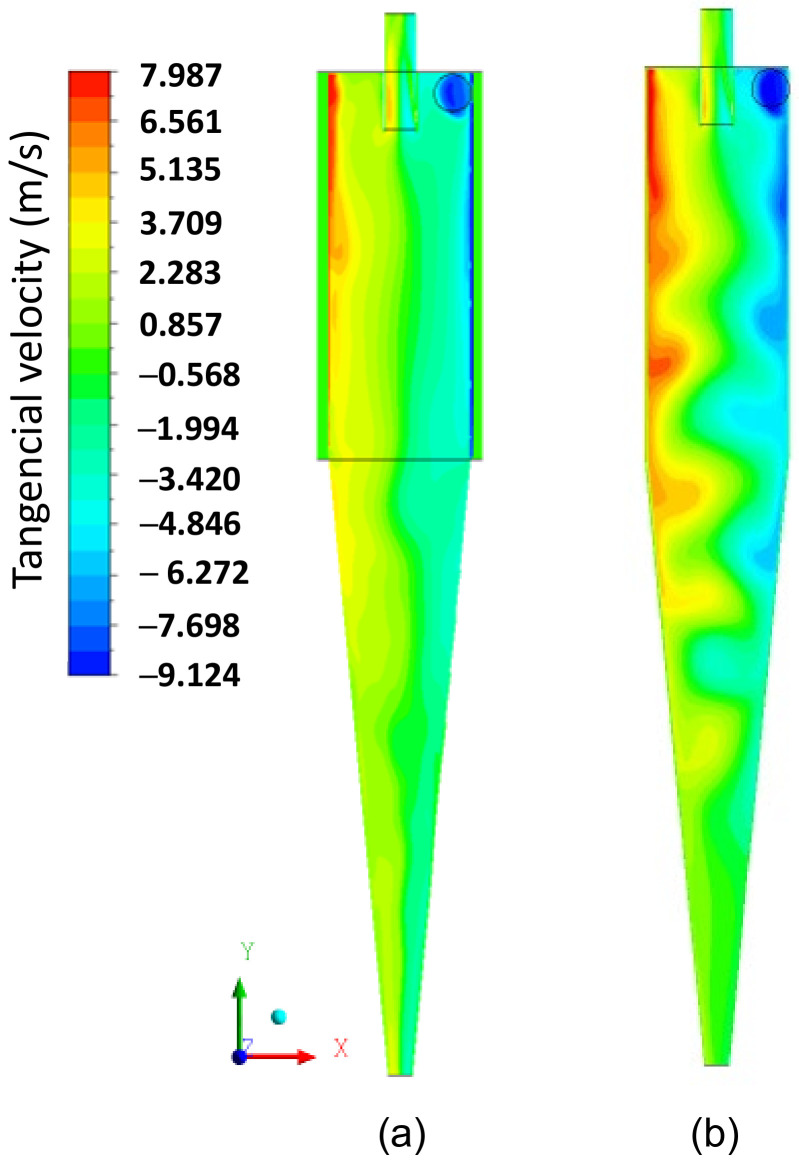
The tangential velocity field on the XY plane for (**a**) the filtering cylindrical hydrocyclone (HciF) and (**b**) the conventional hydrocyclone (Hcon).

**Figure 16:**
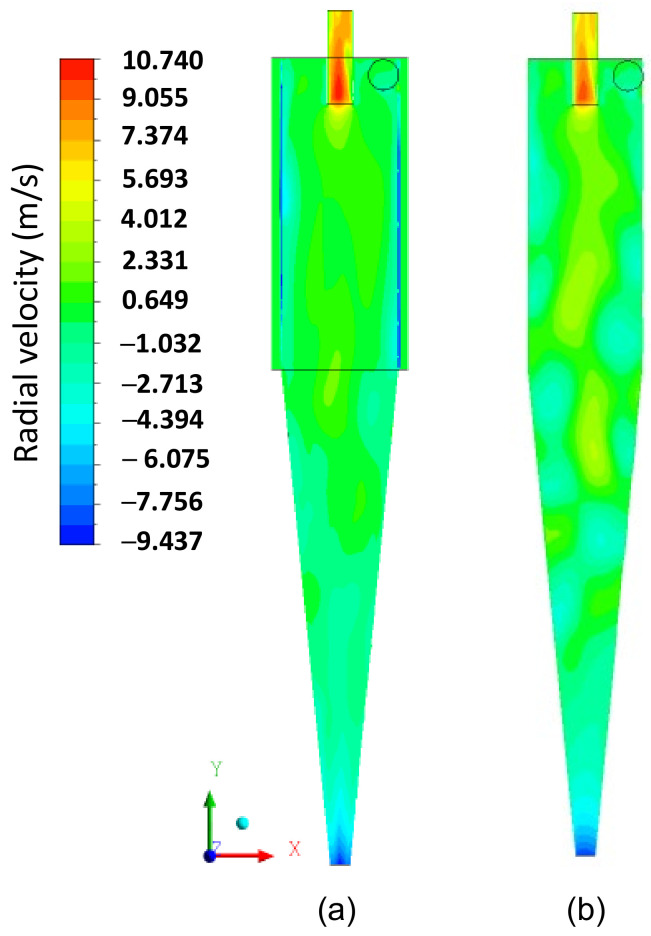
The radial velocity field on the XY plane for (**a**) the filtering cylindrical hydrocyclone (HciF) and (**b**) the conventional hydrocyclone (Hcon).

Figure 14, Figure 15 and Figure 16 present the axial, tangential, and radial velocity fields, respectively, in the longitudinal XY plane for the filtering (HciF) and conventional (Hcon) hydrocyclones. Upon evaluating these figures, it can be observed that the presence of the filtrate flow in the filtering cylindrical hydrocyclone significantly influences the velocity profiles compared to those observed for the conventional hydrocyclone. Regarding axial velocity (Figure 14), several recirculation zones emerge in the absence of the filtering medium. The tangential velocity field (Figure 15) shows a significant decrease in velocity across both the cylindrical and conical regions. Additionally, in the radial velocity field (Figure 16), higher velocities are clearly observed near the walls of the conventional hydrocyclone, as expected. 

The centrifugal field is directly proportional to the tangential velocity and separation efficiency. Therefore, to analyze the tangential velocity, it is necessary to consider the filtrate flow rate, axial, and radial velocities. Examining the behavior of radial velocity, as illustrated in Figure 16, an increase in this hydrodynamic parameter is observed in certain regions in the cylindrical membrane. This phenomenon causes the transport and elevation of the number of particles near the wall of the hydrocyclone, facilitating their discharge through the lower orifice of the hydrocyclone (underflow). This alteration in behavior affected the overall efficiency, with the conventional hydrocyclone achieving a 12% higher efficiency than the filtering hydrocyclone.

**Figure 17:**
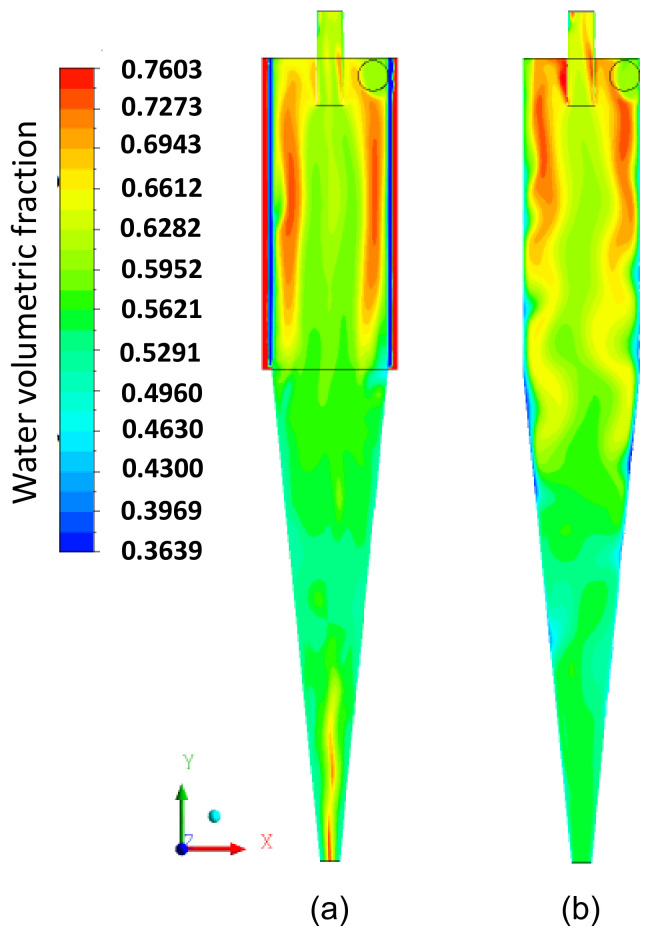
The volumetric fraction field of water on the XY plane for (**a**) the filtering cylindrical hydrocyclone (HciF) and (**b**) the conventional hydrocyclone (Hcon).

**Figure 18:**
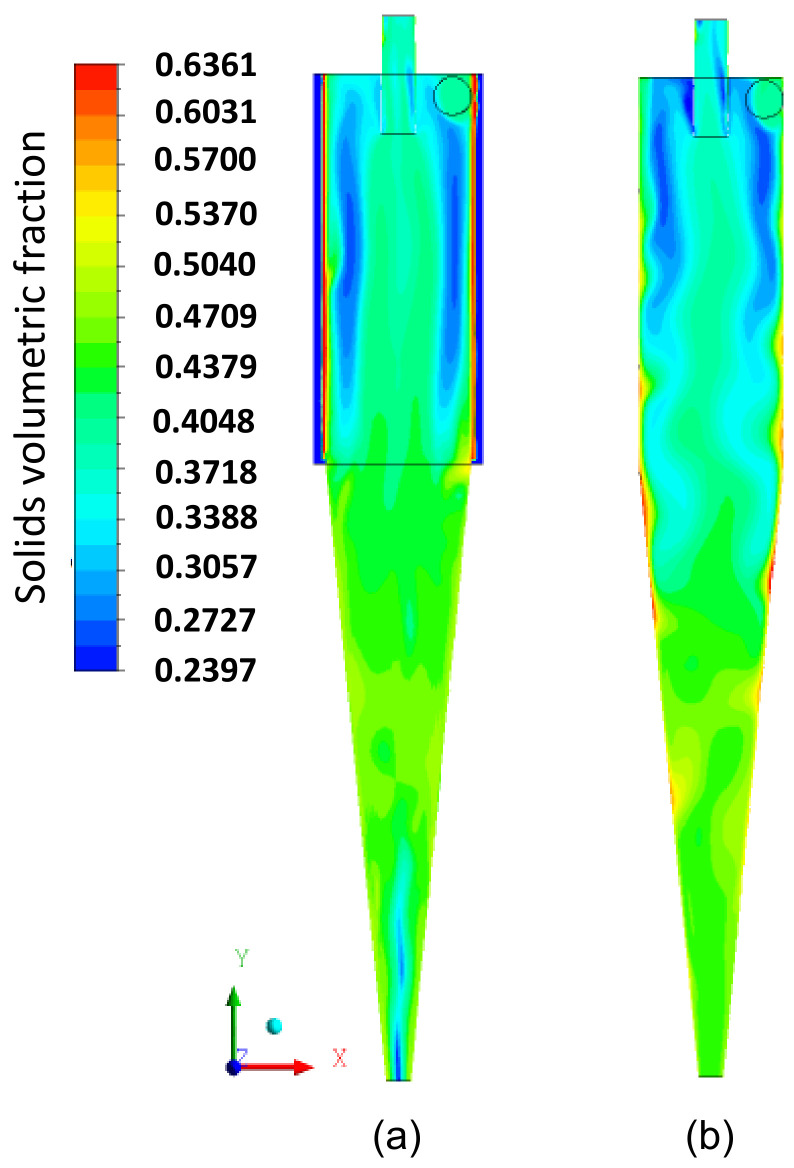
The volumetric fraction field of particles on the XY plane for (**a**) the filtering cylindrical hydrocyclone (HciF) and (**b**) the conventional hydrocyclone (Hcon).

**Figure 19:**
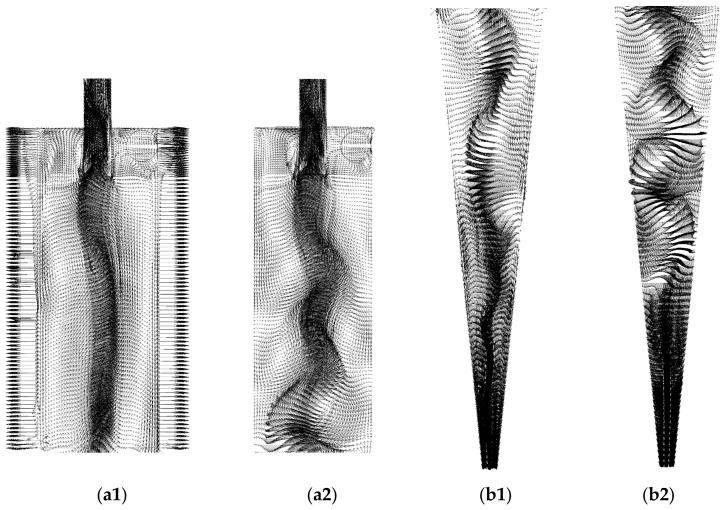
The fluid vector field [m/s] in the cylindrical part on the XY plane for (**a1**,**a2**) the filtering cylindrical hydrocyclone (HciF) and (**b1**,**b2**) the conventional hydrocyclone (Hcon).

Figure 17 illustrates the water volumetric fraction field on the XY plane for the filtering cylindrical hydrocyclone (HciF) and the conventional hydrocyclone (Hcon). Upon analyzing this figure, an increase in the volumetric fraction of water near the central axis is observed near the “underflow” outlet when comparing HciF with Hcon. According to Figure 18, when comparing the volumetric fraction fields of particles on the XY plane for (a) HciF and (b) Hcon, a decrease in the particle fraction near the overflow outlet is observed, reducing the short-circuiting phenomenon (see Figure 19), providing the conventional hydrocyclone with greater efficiency than the filtering cylindrical hydrocyclone. 

### 3.4. Advances and Challenges to Increase the Performance of Filtering Hydrocyclones

The progress and needs to increase the separation performance of the hydrocyclone are related to different parameters, such as inlet particle concentration, inlet mixture velocity, geometry and dimensions, wall roughness, particle diameter, particle density, particle–particle and particle–wall interactions, and many other effects [19,47,48]. Regarding filtering hydrocyclones, the hydrodynamic performance is related to membranes and other fixed parts of the equipment. Then, to complement this information, we can cite the following:

(a) Regarding the membranes: Implementing porous membranes in hydrocyclones involves addressing practical challenges such as construction planning and managing pressure gradients across the device. Effective materials selection, considering mechanical, thermal, and hydric properties like rupture and flexural stresses, thermal conductivity, thermal diffusivity, specific heat, porosity and permeability, is crucial for optimizing filtering hydrocyclone performance and reliability.
(b) Regarding the others fixed part of the filtering hydrocyclone: Construction planning and economic analysis involving the inlet and outlet types, the shape and dimensions of fixed parts, such as overflow, underflow, and cylindrical and conical parts, play an important role in increasing the separation performance of filtration devices.
(c) Regarding numerical simulation: The robustness of the mathematical modeling and advanced and innovative numerical simulation tools can significantly improve the understanding related to the interactions between solid and fluid particles during the isothermal and non-isothermal flows in the filtering hydrocyclone. These powerful tools allow excellent predictions and easy control of physical and thermal effects, ensuring the safety of the filtration device and helping to make safe decisions.

From these comments, it can be seen that there are a large number of opportunities for new research focusing on optimizing the performance of filtering hydrocyclones. Innovations and developments in materials science and more sophisticated simulation tools coupled with a well-designed filtering hydrocyclone structure are essentials for maintaining flow field stability and will be key to improving the viability and separation efficiency of filtration devices.

## 4. Conclusions

The numerical results from the simulation of the water–solid particle separation process in a filtering cylindrical hydrocyclone lead to the conclusion that the mathematical model used effectively assessed the fluid dynamic behavior of the phases within and along the wall of the studied filtering hydrocyclone, being representative of the proposed problem. The simulations revealed higher pressures near the porous membrane, and higher concentrations of solid particles in the conical region, with water being more concentrated in the cylindrical part of the hydrocyclone, due to the equipment’s geometric conditions. Additionally, the simulation results indicated that the volumetric flow rate of the filtrate influences the internal flow dynamics in the hydrocyclone when compared to the results for a conventional hydrocyclone (non-porous cylindrical wall) under the same geometric and operational conditions. The conventional hydrocyclone showed higher pressure gradients near the cylindrical wall and greater separation efficiency, based on the mass flow rate of particles in the underflow, compared to the results obtained in the filtering hydrocyclone.

## Figures and Tables

**Table 1:** Dimensions of the filtering hydrocyclone (HciF).

Dimensions		Hcon	HciF
Inlet diameter (mm)	Di	7.8	7.8
Underflow diameter (mm)	Du	5	5
Cylinder diameter (mm)	Dc	30	30
Overflow diameter (mm)	Do	6.6	6.6
Vortex finder length (mm)	l	12	12
Cylinder height (mm)	h	80	80
Cone height (mm)	H	127	127
Total Length (mm)	L	207	207
Membrane Thickness (mm)	Esp	-	2.5

**Table 2:** Number of divisions on the edges used in mesh refinement.

Nodes											
A	B	C	D	E	F	G	H	I	J	K	L
8	20	2	12	5	10	10	5	20	16	70	80
10	22	3	12	7	12	12	7	22	18	70	87
12	24	4	14	9	14	14	9	24	20	72	92

**Table 3:** Information on the numerical meshes used in the research.

Meshes	Number of Elements	Number of Nodes
01	430,893	405,614
02	664,463	630,643
03	840,341	801,852

**Table 4:** Physical properties of the fluid and solid particle in the study.

Parameter	Material		Source
	Water	Solid Particle	
Density (kg/m^3^)	997	2860	Façanha [28]
Viscosity (Pa s)	8.889 × 10^−4^	---	
Molar Mass (kg/kmol)	18.05	---	
Surface Tension (N/m)	0.01	---	

**Table 5:** Permeability and porosity of porous media.

Parameter	Value	Source
Permeability (m²)	1.71 × 10^−16^	Façanha [28]
Porosity (-)	0.138	

**Table 6:** Operational parameters for the case studied in this research.

Volumetric Flow Rate (cm³/s)	Phase Volumetric Fraction (-)		Fonte
	Continuous	Dispersed	
295.7	0.9	0.1	Façanha [28]

## Data Availability

The raw data supporting the conclusions of this article will be made available by the authors on request.
